# Induced Circular Dichroism From the Binding of Achiral Bivalent Ligands to Transthyretin

**DOI:** 10.1002/jmr.70037

**Published:** 2026-04-24

**Authors:** Stephen Wood, Yiwei Guan, James Pitts, Raj Gill, Simon Kolstoe, Támas Jávorfi, Rohanah Hussain, Giuliano Siligardi

**Affiliations:** ^1^ Division of Medicine University College London London UK; ^2^ School of Biological Sciences University of Portsmouth Portsmouth UK; ^3^ Department of Biological Sciences, Birkbeck University of London London UK; ^4^ Diamond Light Source Harwell Science and Innovation Campus Didcot UK

**Keywords:** induced CD, stabiliser, transthyretin, x‐ray

## Abstract

Achiral bivalent ligands capable of simultaneous occupation of both hormone binding sites of transthyretin generate a substantial and complex induced near ultraviolet circular dichroism spectrum during protein binding, revealing the dynamics of this process. Reduced temperature and pH slow the interaction and reveal two phases consistent with formation of an encounter complex and progression of the interaction through the core of the transthyretin tetramer. The x‐ray structure of the protein‐ligand complex confirms the endpoint of the binding trajectory and shows evidence of plasticity in the structure, with substantial disturbances of some mainchain and sidechains within and adjacent to the binding channel. This study highlights the effective complementarity of CD and x‐ray investigations.

## 1 Introduction

The human plasma protein transthyretin (TTR) is metastable and prone to amyloid fibre formation. Dissociation of the native tetrameric quaternary structure and subunit misfolding is widely reported as the mechanism for this process [1]. Cryo‐electron microscopy of ex vivo fibres shows that the misfolding is radical, with the polypeptide chains adopting a hydrogen bonded stack of a looped planar form [2]. Recent observations suggest that limited proteolysis and shear forces may be important factors in initiating the structure transition [3, 4, 5]. Deposition of amyloid fibres causes tissue damage and disease. Wild type TTR amyloid fibres are frequently deposited in the heart of elderly individuals and have recently been recognised as an important cause of cardiac failure in these individuals. There are over 100 point mutations in the TTR gene which encode TTR variants with reduced stability, leading to autosomal dominant hereditary systemic amyloidosis which can present at any time from early adult life onwards and cause peripheral and autonomic neuropathy, cardiomyopathy, renal failure and blindness due to vitreous amyloid deposition [6].

Although TTR is a relatively abundant protein in human blood and capable of binding two molecules of thyroxine, thyroxine‐binding globulin and albumin are the main carriers of thyroxine (T4) in this tissue and the hormone binding sites of TTR are mostly vacant. The two T4 binding sites of TTR form a substantial channel at subunit interfaces of the tetrameric protein, and their occupation by T4 displaces water molecules and provides inter‐subunit ligand‐mediated interactions. Some success has been achieved in the discovery of thyroxine mimetics that kinetically stabilise the TTR tetramer and are effective as anti‐amyloidogenic agents. Tafamidis, developed by Kelly and coworkers, is a licenced medicine; 2024 FDA‐approved AG10 (acoramidis) [7, 8, 9, 10, 11] and others are in development.

**FIGURE 1:**
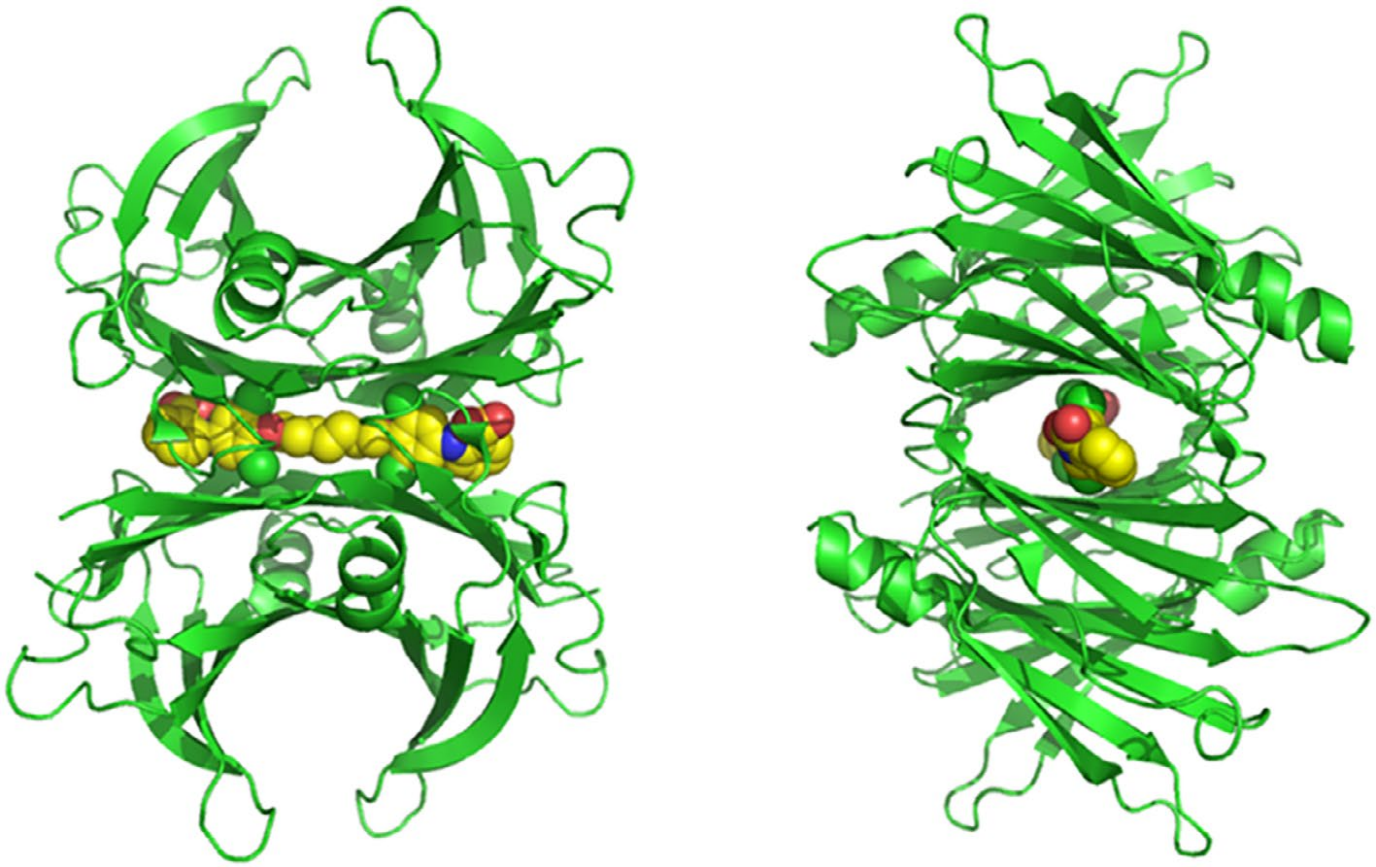
Orthogonal cartoon representations of the TTR tetramer (green ribbons) showing the position of the bivalent ligand mds84 (carbon‐yellow, chlorine‐green, nitrogen‐blue, oxygen‐red) in the binding site (PDB 3IPE) [12] prepared with PYMOL.

The serendipitous discovery of a symmetrical bivalent chlorinated bis‐aryl ligand (mds84) capable of binding to both thyroxine binding sites of native TTR at the same time and with much higher affinity than monovalent compounds like tafamidis provided a significant change in outlook for the development of TTR stabilisers [12, 13, 14, 15]. Furthermore, such compounds appear to provide some protection against proteolysis [4]. The x‐ray structure of the mds84‐TTR complex obtained by co‐crystallisation showed the clear occupation of both hormone binding sites but indicated that there would likely be considerable steric barriers to ligand entry by threading through the centre of the TTR tetramer (see Figure 1).

Earlier work had already shown that other bivalent compounds only bound if the TTR tetramer was denatured and reassembled in the presence of ligand [16]. For mds84, this was not required, implying that some relaxation or partial disassembly of the tetramer enabled binding. It is known that the TTR tetramer is a dynamic assembly capable of intermolecular subunit exchange [17], marked negative cooperativity for binding of the second molecule of T4 [18] and normal mode analysis indicates that a reciprocal iris‐like opening and closing motion of the hormone binding pockets is feasible [19]. These processes may be important contributors to the binding of bivalent ligands to TTR.

**FIGURE 2:**
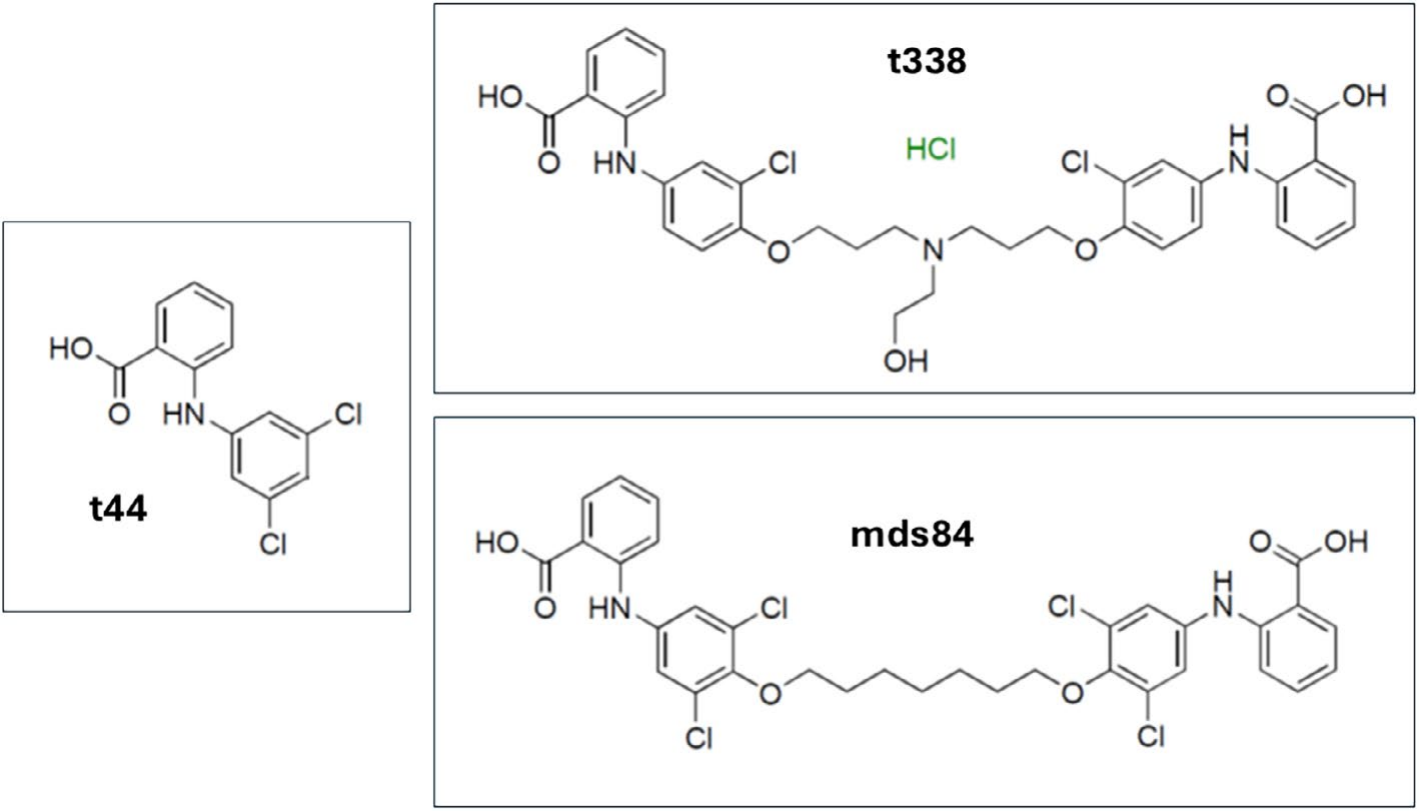
Chemical structures of t338, t44 and mds84 ligands employed in this investigation.

To further understand the binding of bivalent ligands to TTR we have measured the induced circular dichroism that arises from the binding process in solution. We have also investigated the x‐ray structure of TTR‐ligand complexes formed by co‐crystallisation and soaking of preformed TTR crystals where protein flexibility is substantially restricted. Parallel experiments were carried out with a head group sub‐structure of mds84 (t44). We had determined the crystal structure of this compound in complex with TTR previously and showed that the orientation was the same as that observed when the substructure was part of mds84 [20]. The structure and spectroscopic effects for binding of an analogue of mds84 that was generated during screening proved particularly informative. This ligand (t338) differs from mds84 in that there is a single chlorine atom on each of the internal aromatic rings and a short nitrogen linked branch in the middle of the linker region (Figure 2). It appears to bind to TTR much more slowly than mds84. These ligands mds84, t338 and t44 are all achiral compounds providing particularly informative induced CD spectra when bound to TTR as the free forms are devoid of any CD signal and this has allowed us to monitor the dynamics of these binding processes.

## 2 Methods

Mds84, t388 and t44 were kindly provided by Mark Bamford (GSK) Stevenage, UK. TTR purified from human blood was purchased from SciPac Ltd., Sittingbourne, UK. and crystallised from 100 mM HEPES buffer pH 6.5 including 200 mM CaCl_2_ by PEG 400 precipitation. Crystals were vitrified directly in mother liquor by plunging into liquid nitrogen. x‐ray data was collected at Diamond Light Source MX stations iO3, iO4‐1 and processed with the Xia2 pipeline [21]. Structure solution and refinement was carried out with CCP4 programmes [22] including Phaser [23], Refmac5 [24] and Coot [25].

CD spectra were recorded on beamline B23 for synchrotron radiation circular dichroism (SRCD) at Diamond Light Source [26]. Typically, spectra were recorded with a rectangular cell of 1 cm path containing 350 μL of buffered protein solution, and volumes of 1–2 μL of ligand dissolved in DMSO were added with rapid manual mixing (10–15 s). The cell path for the t44 titration was 5 mm. The protein concentration in each experiment was in the region of 1 mg/mL (about 18.2 μM as tetrameric form). Spectra were recorded in 10 mM Tris/10 mM NaCl buffer at pH 8 and in 10 mM phosphate buffer at pH 6.5. Measurements at 4°C and 20°C were carried out using a temperature‐controlled Peltier cuvette holder (Quantum Northwest Inc., USA). An incubation of 10 min was applied for static temperature measurements. For time‐course measurements, temperature rate was 100 °C/min, reaching 20°C in about 10 s.

The CD titration was carried out by adding to the cuvette of 5 mm pathlength containing 1500 μL of TTR tetramer solution (13.7 μM) nine consecutive 6 μL aliquots of stock solution (1.142 mM) of the ligand, each corresponding to a 0.33 M equivalent. The titration spectra were corrected for dilutions, and CD data were processed and analysed using the CDApps suite of software programmes of beamline B23 [27].

## 3 Results

### 3.1 Induced CD

Since all ligands shown in Figure 2 are achiral, they do not exhibit circular dichroism. All ligands possess the same chromophore moiety absorbing in the near‐UV region (250–400 nm) whilst the link between the two chromophore heads in mds84 and t388 is transparent. This was confirmed by recording control spectra in the near UV region for ligand diluted into buffer alone in each experiment below.

**FIGURE 3:**
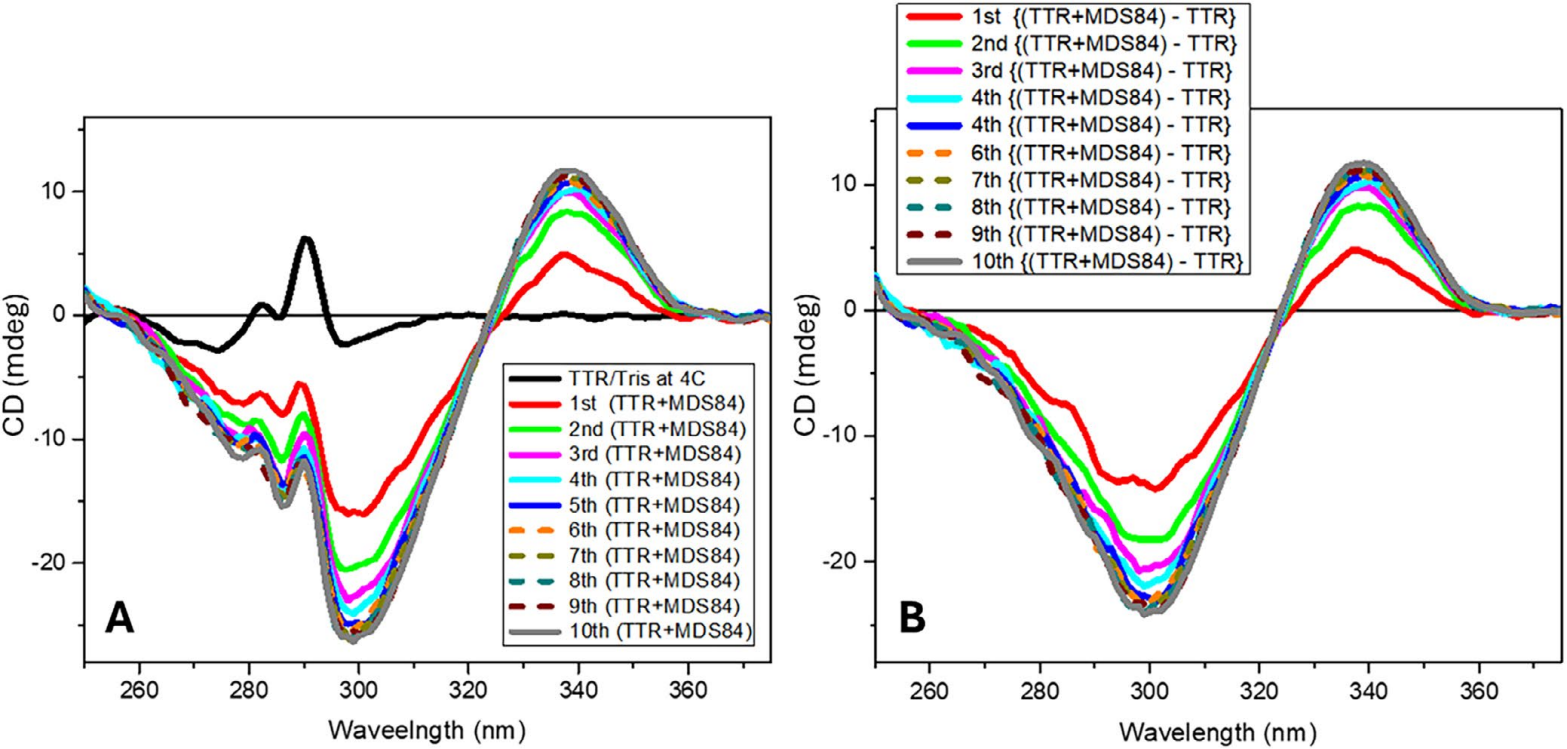
(A) Ten repeated consecutive near‐UV SRCD spectra of TTR (1 mg/mL) following addition of two equivalents of mds84 at pH 8, 4°C. The pathlength was 1 cm and 1–2 μL of ligand stock dissolved in DMSO was rapidly mixed with 350 μL of protein solution. (B) Difference spectra calculated by subtracting the spectrum of TTR (black spectrum in (A)) from subsequent recordings to illustrate the induced SRCD component during ligand binding. The induced spectral changes was completed in about 15 min corresponding to the fifth spectrum (blue). Each spectrum was scanned in about 3 min.

The CD spectrum of TTR (1 mg/mL in 10 mM Tris‐HCl buffer pH 8, 10 mM NaCl) in the near UV region (250–380 nm) shows two sharp positive bands at 290 and 280 nm superposed on a broad negative band centred at 280 nm (Figure 3A). The largest band at 290 nm likely derives from tryptophan sidechains while those at about 280 nm derive from tyrosine sidechains. The spectrum is unchanged between 20°C and 4°C and has been reported in previous studies [28, 29]. It is included as the baseline measurement in all the following results.

The addition of a twofold molar excess of the achiral bivalent ligand mds84 to the same TTR solution at 4°C revealed the formation of an induced (+/−) bisignate spectrum with a positive band centred at 340 nm and a negative band at 300 nm (Figure 3A,B). The full extent of both bands was not reached until completion of five repeated scans over 15 min. Essentially the same final spectrum was generated at 20°C but in a much shorter time (not shown). The induced spectrum derived by subtracting the protein spectrum from that of the protein‐ligand complex (Figure 3B) may include features that arise from the bound ligand and from perturbation of aromatic protein sidechains. As TTR does not possess any chromophore that absorbs UV light above 300 nm, the induced CD band at about 340 nm is entirely due to the bound ligand chromophore. At 300 nm, although there is a strong electronic transition in the UV spectrum of the ligand, it is not possible to distinguish between CD contributions from the ligand and aromatic protein sidechains. However, changes in both spectral regions report on the binding process. The induced CD, unambiguously indicative of the ligand binding interaction, shows the end stage of one relatively slow component of the interaction of the bivalent ligand with TTR. The crystal structure of the complex described previously [12] indicates that the end‐product of this process corresponds to the location of one bis‐aryl component of the bivalent ligand bound to each of the hormone binding pockets of the TTR tetramer.

**FIGURE 4:**
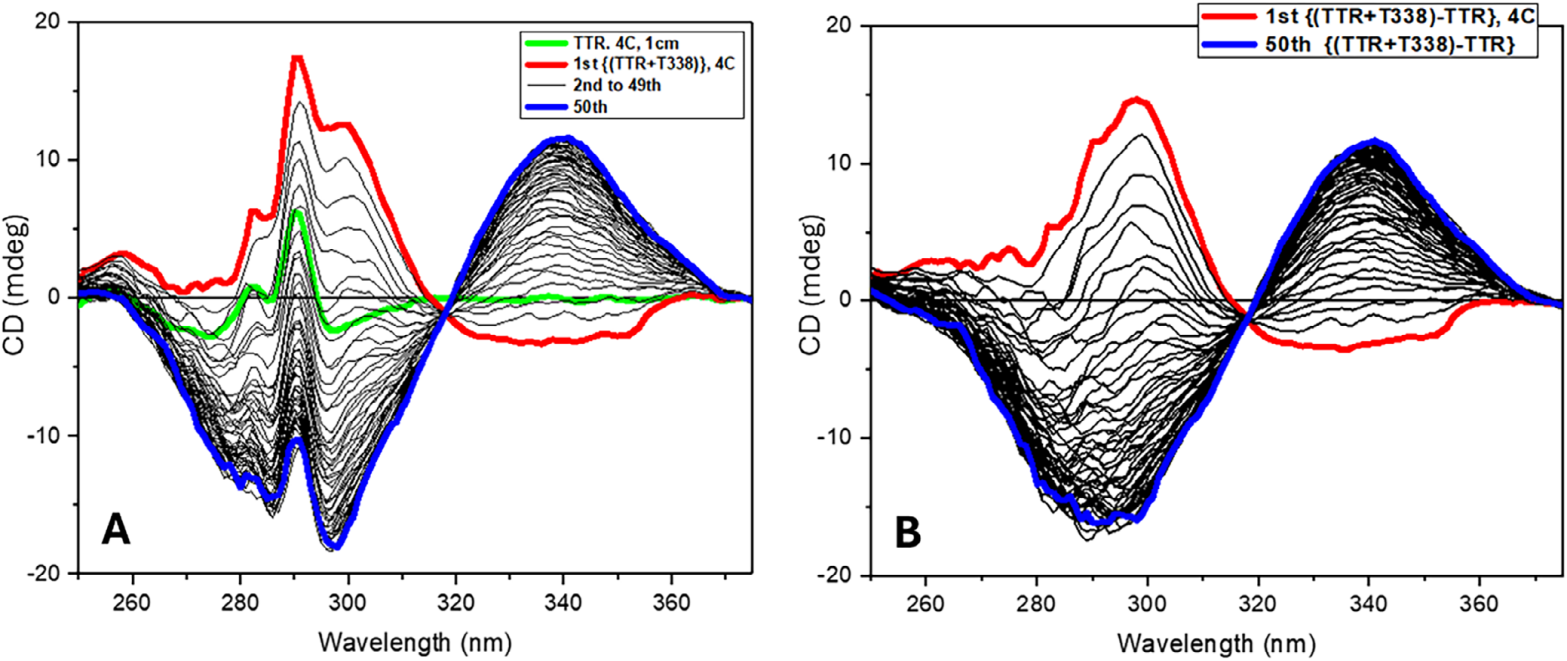
(A) Fifty consecutive repeated near‐UV SRCD spectra of TTR with two equivalents of ligand T338 at 4°C measured under conditions as in Figure 3. (B) Difference SRCD spectra calculated by subtracting the spectrum of TTR (green) from each consecutive repeated spectrum of TTR + T388 mixture indicate the ligand binding of achiral T388 to TTR as a function of time as each spectrum was scanned in about 3 min. The blue spectrum was the first measured of the TTR + T338 (1:2) complex and the spectral changes were completed in about 150 min as shown by the 50th spectrum (red).

When the same experiment was carried out with the bivalent ligand t338, the first spectrum was again bisignate but of opposite sign (−/+) to that described above for mds84, negative at about 340 nm and positive at about 300 nm (Figure 4A,B). During the following consecutive repeated scans, a gradual change of the sign of the bisignate induced CD from negative–positive (−/+) to positive–negative (+/−) was observed, becoming like that of mds84 (Figure 3) over 150 min. These spectra indicate the transition from an initial interaction state to a final one; relatively rapid formation of some intermediate with a (−/+) bisignate spectrum whose formation was too fast to observe in our simple experimental procedure and its very slow transformation to a final state with a (+/−) bisignate spectrum. The similarity of the final spectrum with that for mds84 binding to TTR and the crystal structure definition of the TTR‐t338 complex (see below) suggest that the same binding interaction mechanism occurred, but that the reduced rate enabled detection of an earlier intermediate in the binding process that had the induced CD spectral signature of opposite sign. This is the result of the bis‐aryl head of t338 and mds84 entering the TTR binding site tunnel with the opposite orientation than that of t44 but reaching the same orientation once entered completely in the TTR tunnel (see cartoon of State 1 and State 2 in Figure 6A,B).

**FIGURE 5:**
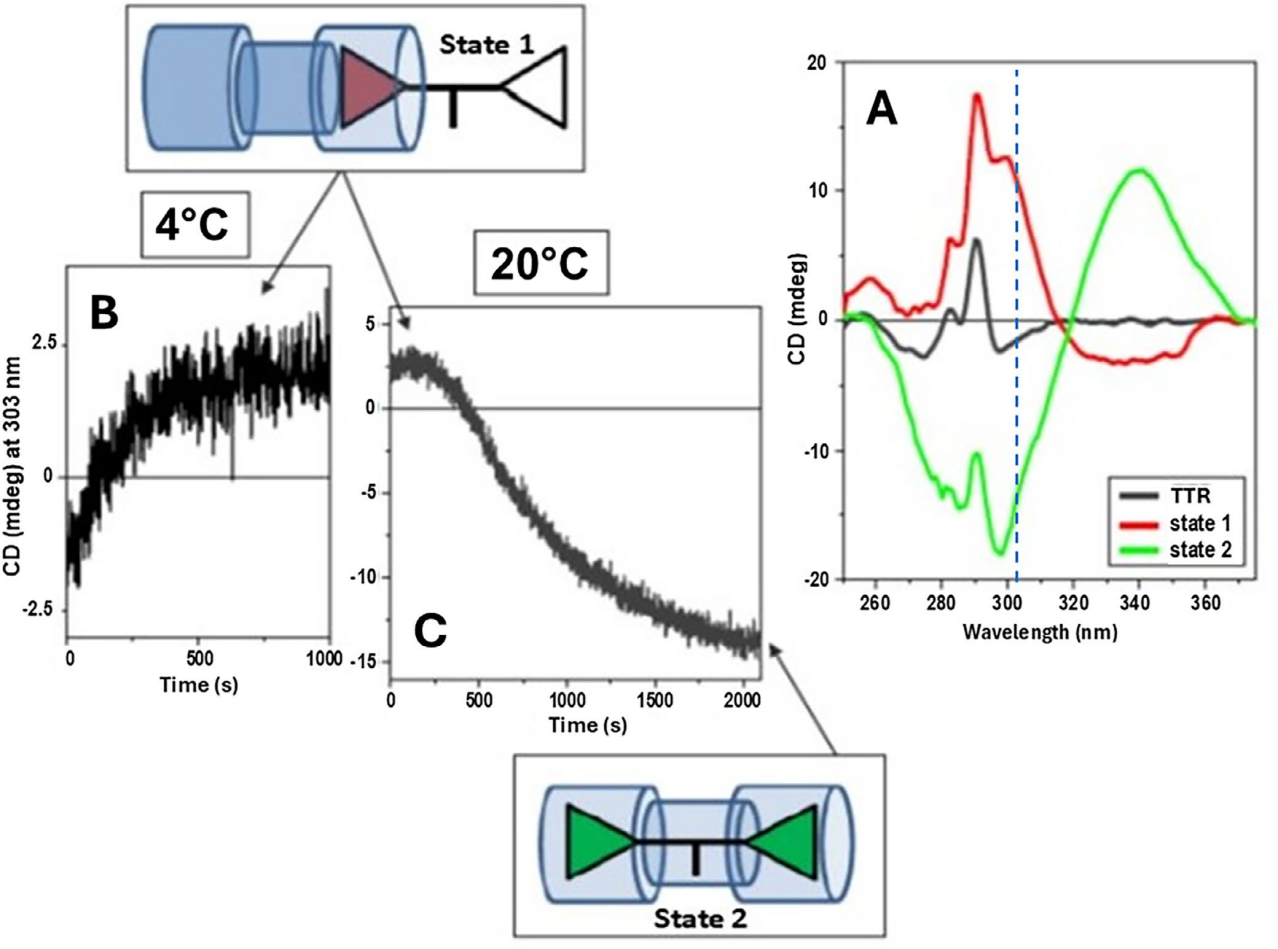
(A) Near‐UV SRCD spectra at 4°C of TTR tetramer (black), achiral T338 ligand bound to the channel of the tetrameric TTR on State 1 (red spectrum represented by the cartoon with red triangle for the partially inserted ligand), and on State 2 (green spectrum represented by the cartoon with green triangles for the complete insertion of the ligand in the TTR channel). (B) Induced CD at 303 nm measured at 4°C as a function of time reaching the plateau for State 1 in 16.6 min (1000 s). (C) State 2 reached in 35 min (2100 s) when the temperature was raised from 4°C to 20°C. In the cartoon, the blue cylinders represent the two binding sites of the TTR tetramer and the connecting channel.

**FIGURE 6:**
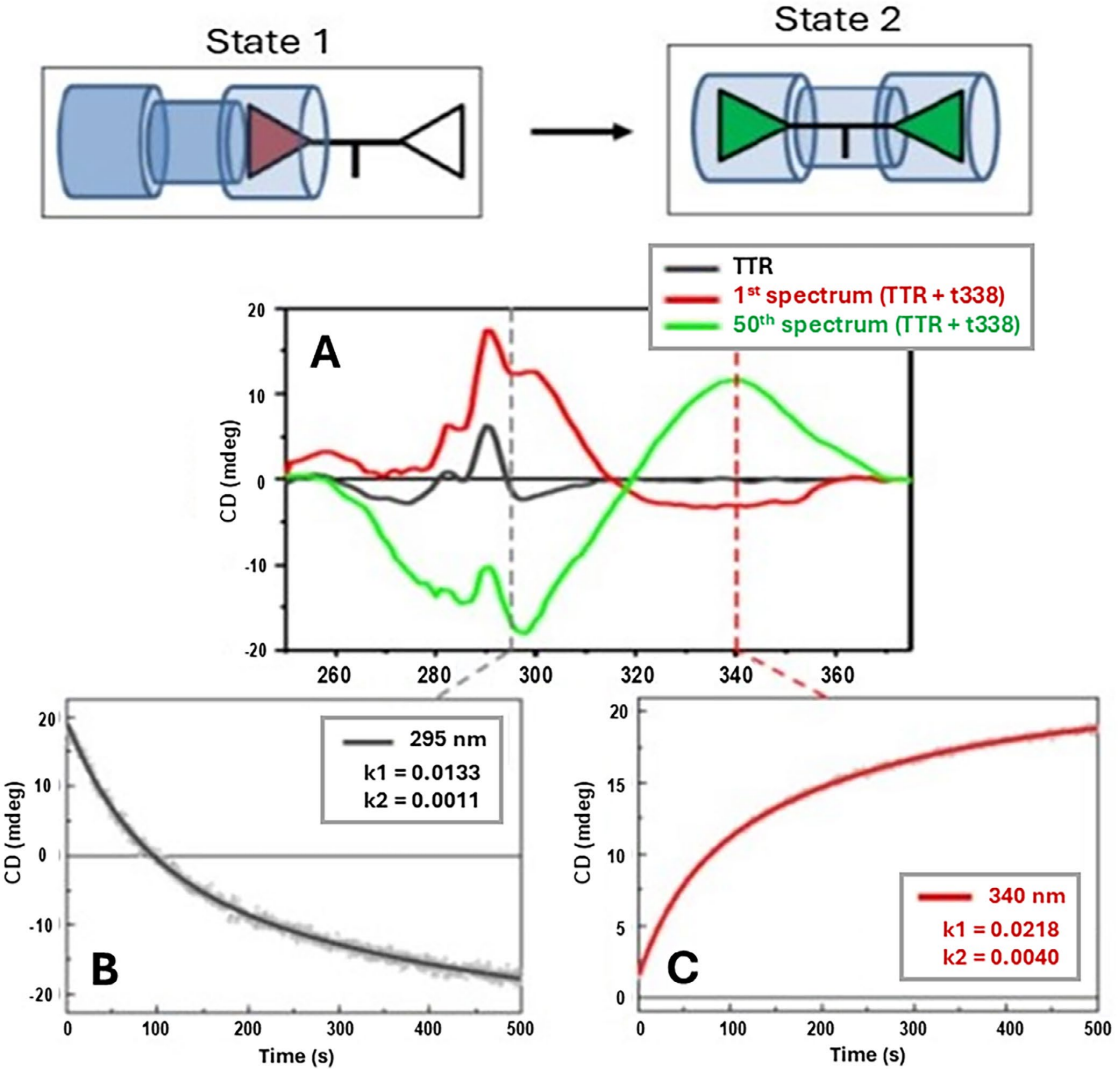
(A) Near‐UV SRCD spectrum of TTR in 20 mM phosphate buffer, pH 6.5, at 4°C (black), first SRCD spectrum of T338 added to TTR (TTR + T338, red) corresponding to State 1, which is T338 entering the TTR tetramer tunnel, and 50th consecutive repeated spectrum of (TTR + T338) (green) corresponding to State 2, which is T338 completely inserted into the TTR tetramer tunnel. The cartoons on the top represent State 1 with one partially inserted aryl chromophore (red triangle) and State 2 with the fully inserted ligand with both aryl chromophores (green triangles). (B) Induced SRCD at 295 nm (black) and (C) induced SRCD at 340 nm (red) for achiral ligand T338 added to TTR and measured as a function of time (1000s). Both (B and C) monitored the evolution of the induced SRCD from State 1 (top left cartoon with red triangle) to State 2 (top right cartoon with green triangles) as a function of time. The fitting of the data using an exponential decay equation (OriginLab) revealed a faster rate of induced SRCD at 340 nm (*k*_1_ = 0.0218, *k*_2_ = 0.0040) than that at 295 nm (*k*_1_ = 0.0133, *k*_2_ = 0.0011).

A further repetition of this experiment in phosphate buffer at pH 6.5 and 4°C showed the gradual growth of the positive CD band in the difference spectrum at 300 nm as observed above (Figure 4A, red spectrum), reaching and holding its maximum intensity after approximately 30 min of repeated consecutive scans. Stepping the temperature of the sample cuvette holder to 20°C during continued scanning led to the decline of this positive band and the gradual development of the (+/−) bisignate spectrum observed previously (at pH 8, 4°C), over more than 60 min. A repeat of this experiment but by recording the CD signal at single selected wavelengths of 295 and 340 nm reaffirmed these observations of the dynamics of the repeated consecutive scans (Figures 5A–C and 6A,B). Fitting of the time‐course of CD changes following the temperature ramp with a two‐phase exponential function (ExpDec2 of OriginLab) revealed that the CD changes at 340 nm (*k*_1_ = 0.0218 and *k*_2_ = 0.004) were faster than those at 295 nm (*k*_1_ = 0.0133 and *k*_2_ = 0.001), being associated with the ligand t338 in State 1 entering the tunnel of the TTR tetramer and moving to State 2 (Figure 6A,B). This is consistent with the positive ICD band at about 340 nm that is only due to the t388 bis‐aryl chromophore whereas the negative ICD at 295 nm also includes the CD contributions of the aromatic side‐chain residues that can sense the crossing of half of the t338 ligand molecule through the TTR tetramer tunnel to reach State 2 (Figure 6A,B).

These CD measurements are consistent with a model of ligand binding where a stable encounter complex is trapped at pH 6.5, 4°C involving a portion of the ligand t338 engaged with the TTR binding site (Figure 5A–C, State 1). The extent of this initial engagement and the number of binding sites involved is unclear, but mass spectrometry did not report binding of two ligands in earlier work employing analogous bivalent ligands [12]. It is possible that the encounter with one site down‐regulates the affinity of the other site as observed with binding of thyroxine to TTR. Although the encounter gives rise to a relatively simple induced CD around 300 nm compared to that of the fully engaged ligand, we suspect that this corresponds to binding of a complete head group of the bivalent ligand. There are no reports from screens of alternate headgroups with lesser degrees of penetration. The stability of such a complex would derive in part from occupation of the outer halogen binding pockets, defined previously from the crystal structure of the thyroxine‐TTR complex, by ligand chlorine atom(s). Raising the temperature to 20°C enables gradual progression of the binding process and generation of an induced CD signal corresponding to the engagement of both binding pockets. The disparity in rates of evolution of the induced CD at 340 nm and 295 nm may be due to slower adjustment of the protein structure to the binding process or possibly the earlier engagement of two copies of the anthranilic acid component of the ligand within the binding channel.

**FIGURE 7:**
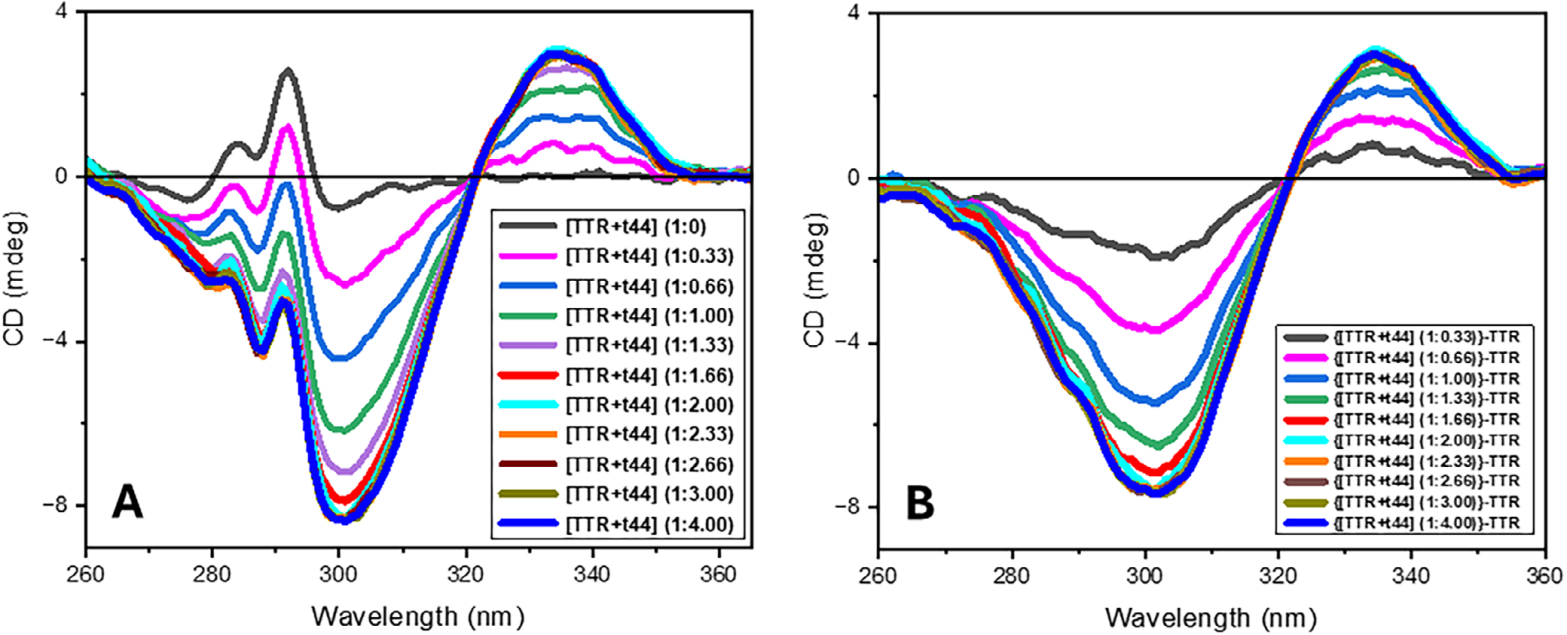
(A) Near‐UV SRCD spectra of the TTR tetramer with incremental addition of n molar equivalents of T44 [(TTR(1) + T44(*n*))] at pH 8, 4°C. pathlength 5 mm. (B) Difference SRCD spectra of the binary complexes calculated by subtracting the spectrum of TTR (1‐black) from those of the {TTR(1) + T44(*n*)}.

Addition of t44 to TTR solution at pH 8 and 20°C immediately generated a qualitatively similar (+/−) bisignate spectrum (Figure 7A,B) as observed at the end‐state of binding of the bivalent ligands mds84 and t338 (Figures 3A,B and 4A,B). This implies that the heads of the bivalent ligand mds84 and t388 are bound in the channel of the TTR tetramer in a very similar orientation and location to that with t44 because the profile of induced CD of the bis‐aryl chromophores in terms of sign and intensity arises from the chiral environment of the TTR binding sites. Sequential sub‐stoichiometric additions of t44 to TTR showed an incremental growth of the bisignate spectrum that reached a plateau with 2 M equivalents (Figure 7A,B). The maximum amplitude of the induced CD was very similar to that observed with mds84. This plateau was not exceeded with a considerable excess of ligand (X20) but higher levels were limited by solubility. These observations are consistent with the occupation of both sites by t44 without cooperativity in accord with prior observations for this class of ligand [30]. In principle it is possible to measure the dissociation constant for the interaction from this mode of titration. However, with ligand affinities in the nM range, a much‐reduced concentration of TTR (2 μM) would be required and proportionately longer pathlength to obtain comparable absorbance changes. Some degradation of the signal/noise ratio would be inevitable. In the current format we can only estimate that the *K*_d_ is in the low nM range in accord with previously reported isothermal titration calorimetry measurements [29].

### 3.2 Crystal Structures

**FIGURE 8:**
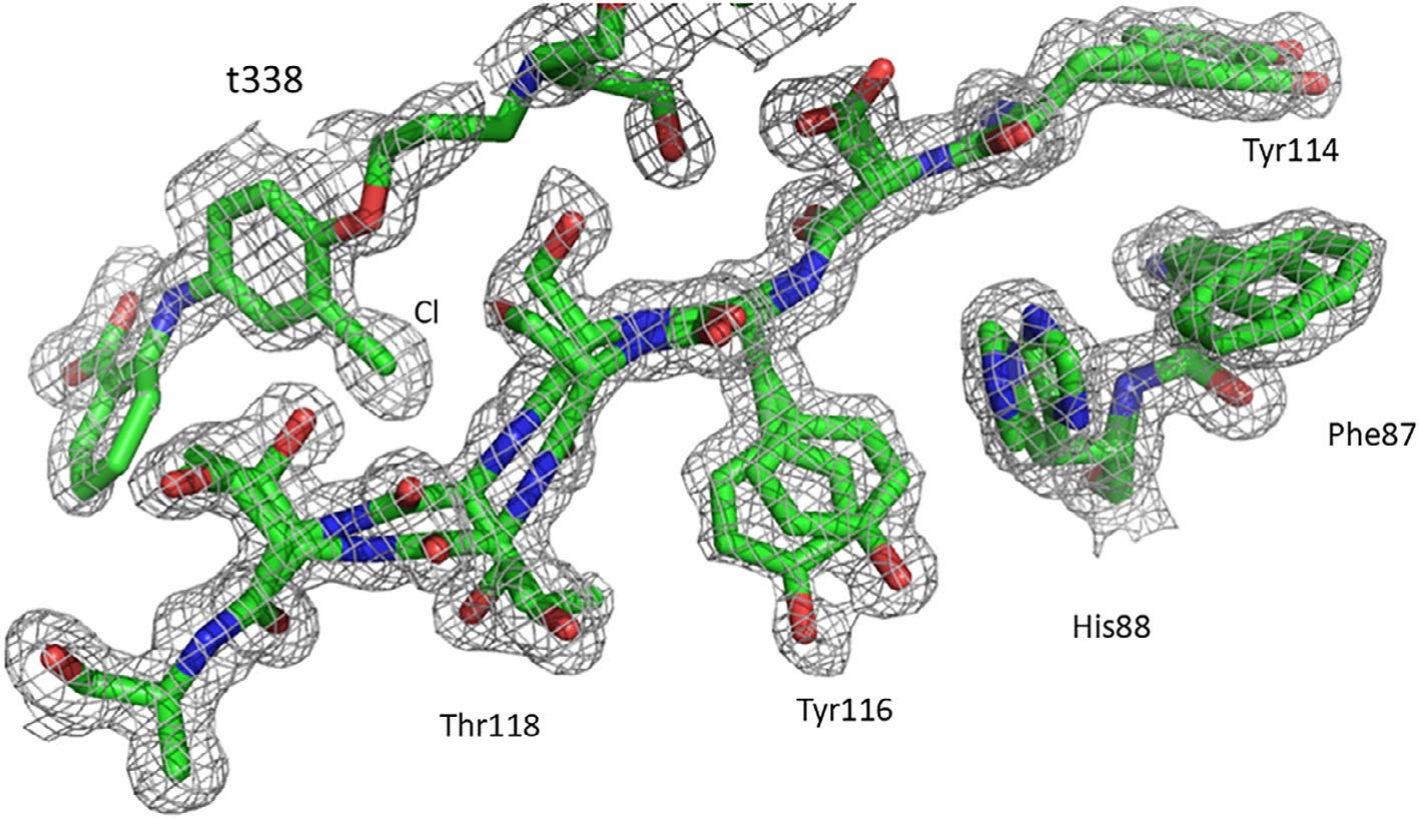
Selected strand and sidechain displacements observed on binding of t338 to TTR tetramer. 2mFo‐DFc electron density at 1.5*σ* and carved at 2 Å in Pymol [31] shows the close approach of a ligand chlorine to the mainchain at Thr118, displacing it and altering the positions of Tyr116 and its neighbours. The displacements are observed as dual conformations.

Co‐crystallisation and soaking procedures were employed to investigate the structure of t338 bound to TTR and of intermediates in the binding reaction. Co‐crystallisation at 20°C, pH 6.5 with excess ligand provided an electron density map at high resolution (1.24 Å) showing clearly that the head groups of t338 engaged both hormone binding sites of TTR in much the same way as mds84 described previously [12] and shown in Figure 1, providing support for understanding the induced CD discussed above (Figure 8). The summary of the x‐ray data statistics is included in Table 1. Although the two structures overlay closely (RMSD 0.25 Å over 1500 atoms), there are some complex novel features in the t338 map that may be of wider interest. The complexity derives to some extent from the fact that the TTR tetramer is a dimer of dimers, with one dimer in the asymmetric unit of the commonly observed orthorhombic crystal form described here. It defines two subunits A and B that are related by a local two‐fold axis and an extended beta sheet arrangement spanning the dimer. The TTR tetramer is generated by a crystallographic twofold axis where A′ and B′ subunits define a second dimer and subunits A, A′ and B, B′ from each dimer form the ligand site at their interface. During refinement, ligands that reside in the thyroxine binding sites are bisected by the crystallographic two‐fold axis and are pragmatically set at 50% occupancy. Electron density maps display two superposed conformations, and it is not clear how these species might be represented in the free tetramer. For t338, the inner aryl rings carry single chlorine substituents which could engage subunits A and B or A and B′. The model we display has the latter arrangement, providing the most crosslinking interactions. Random incorporation of such tetramers in crystal growth would establish the ambiguity. The chlorinated rings in t338 are tilted and located slightly closer to the centre of the tetramer so that the distance between equivalent chlorine sites is 1.5 Å shorter than in the mds84 complex reported earlier. The repositioning of the chlorine atoms is associated with a displacement of the nearby main chain of Ser117/Thr118 of ~1 Å in both subunits that gradually dissipates on either side, but the disturbance is sufficient to generate small adjustments of a range of sidechains including Tyr116, His88, Phe87 and Tyr114 and reorganisation of their interactions (Figure 8). In all cases the electron density is modelled as dual conformations. These sidechains reside on the β‐strands that enclose the binding site but are oriented away from the site. The adjustments also extend to Glu92, Ala91, His90 that populate the local symmetry axis of the TTR dimer in the asymmetric unit. The path of the alkyl linker was buckled at the centre to accommodate the repositioned chlorine atoms, and the hydroxy‐ethyl branch displaced some and engaged other water molecules that reside at the tetramer core. The electron density was relatively weaker in this region. While large regions of the map were very good, the dual conformations of some main chain and sidechain regions were difficult to model without steric clashes. Collectively these observations suggest that two forms of TTR subunit reside in the crystal, one more closely resembling the apo‐protein and the other showing a host of displacements where the chlorine substituent has perturbed Thr118.

**TABLE 1:** x‐ray diffraction data for co‐crystallised TTR‐t338 complex using beamline I04‐1 Diamond Light Source.

Wavelength (Å)	0.9174
Space group	P2_1_2_1_2
Cell dimensions *abc* (Å)	85.43, 43.66, 64.67
Reflections measured	893,801 (60,603)
Reflections unique	69,058 (4926)
*R*_free_ set	3465
Redundancy	12.9
*d*_max_/*d*_min_ (Å)	85.43–1.24
Completeness (%)	99.6
*R*_sym_ ^a^	0.059 (2.05)
*R*_meas_ ^a^	0.064 (2.25)
*R*_pim_ ^a^	0.018 (0.628)
Average (*I*/*σI*)	17.5 (1.3)
CC_1/2_ ^a^	1 (0.6)
Wilson *B* factor (Å^2^)	15.53
*R*_work_ (%)	14.9
*R*_free_ (%)	18.7
RMSD bonds (Å)	0.033
RMSD angles (^o^)	2.786

*Note:* Values in parenthesis are for highest resolution shell. Coordinates deposited with PDB code pdb_00009ry5.

^a^ Terms fully defined in Reference [32].

Extended soaking of TTR crystals in mother liquor containing 1 mM t338 at pH 6.5, 20°C produced essentially the same structure as that obtained from co‐crystallisation, but with reduced occupancy (Figure File S1). When the soaking time was limited to 1 h, the clear but relatively weaker difference density within the hormone binding pocket no longer provided interpretable evidence of the ligand orientation. When crystals were pre‐soaked in mother liquor containing 15% (v/v) DMSO to achieve higher concentrations of t338 during the soaking interval, the new density in the binding site took on a different but equally uninterpretable form. These observations may arise from the superposition of progressive phases of ligand engagement as observed by CD spectroscopy but were too weak and complex to justify building definitive models and x‐ray refinement.

Soaking preformed crystals of TTR in mds84 containing mother liquor at room temperature prior to vitrification and x‐ray data collection showed essentially the same result as described previously for co‐crystallised materials [12]. However, the limited solubility of mds84 in aqueous solution required much longer soaking times than were effective with t338 and the ligand occupancy was lower. Nevertheless, the electron density could be fitted with both bis‐aryl head groups positioned with their halogen atoms towards the inner part of each hormone binding pocket, connected by the linear alkyl chain through the core of the TTR tetramer (Figure S1). As observed with t338 soaking, there was no evidence of crystal cracking or other deterioration during the soaking procedure, and the crystals remained highly ordered, suggesting little if any structural change in the protein that might be required for ligand binding was transmitted to lattice contacts.

x‐ray data obtained by soaking T44 into preformed TTR crystals for short periods of time (1–2 h) showed weak but unmistakable difference electron density for the ligand with chlorine atoms towards the inner part of the binding site and corresponding to the orientation observed by co‐crystallisation [19] and with mds84 [12].

## 4 Discussion

The crystal structures of mds84 and t338 in complex with TTR obtained by co‐crystallisation or soaking preformed protein crystals in the corresponding ligands show the ligands fully engaged with both binding sites. Therefore, a portion of the ligand must have threaded through the core of the TTR tetramer in the crystal lattice to reach its final resting position. This process does not impact the crystal packing that depends upon the integrity of the protein surface to sustain lattice contacts. Gross dynamical aspects of the TTR tetramer assembly noted above are therefore unlikely contributors to the binding process. The generally lower occupancy of soaked complexes is not unexpected in view of the size, extended shape and low solubility of the ligands that must permeate the solvent channels of the crystal to reach the binding site.

The encounter complex observed by CD spectroscopy for the binding of t338 at 4°C, pH 6.5 most likely involves engagement of a single bis‐aryl headgroup in an orientation opposite to that of the mature complex. Therefore, during progression of the binding process, the orientation of one of the bis‐aryl head groups must invert as illustrated in Figures 5 and 6. This is in accord with the bisignate induced CD spectrum (negative band at about 340 nm and positive band at about 300 nm) of the achiral ligand head entering from one side of the chiral channel of the tetrameric TTR acquiring an opposite induced chirality on reaching the other side of the TTR channel. A similar trajectory is probably followed in the binding of mds84 as the induced CD and the crystal structures of the endpoint are very similar.

The ligand t44, which is the main chromophore moiety of msd84 and t388, clearly prefers to bind TTR with its chlorinated ring towards the tetramer core during co‐crystallisation or soaking but must be able to bind in a reverse mode in the mds84‐TTR encounter complex in solution and in the crystal. This ability of TTR ligands to bind in forward and reverse modes has seriously complicated some earlier structural investigations of complexes when both ligand orientations are bound at the same time. This complication is magnified by the positioning of the ligands along a crystallographic two‐fold axis and is especially difficult if the ligand lacks prominent marker atoms like chlorine. There are, however, clear examples of analogous bis‐aryl ligands binding in reverse mode [30, 31, 33] (PDB code 3CN4). Thus, it appears that the t44 molecule binds preferentially in forward mode but will also bind in reverse mode with lower affinity. The higher affinity interaction of the forward mode must be a major contributor to the stability of the final complexes formed with bivalent ligands where two copies of the head group are simultaneously engaged, and the interaction is effectively irreversible [12].

The majority of the ligand–protein interactions observed for bound mds84 and t338 are non‐polar. However, the location of Lys15 at the entrance of the ligand binding site of the protein and the failure of carboxymethylated mds84 [12] to bind suggests that an electrostatic interaction between these two groups may be an early feature in the formation of the encounter complex. This cannot be achieved for t44 binding in forward mode and is consistent in the final complex with both ligand carboxylates in close proximity to Lys15. Full engagement of the head group t44 in reverse mode would bring the charged ligand carboxylate into a site that in forward mode binds a halogen atom. The carboxylate would also be within hydrogen bonding distance of Ser117 at the core of the tetramer and potentially perturbing the complex network of waters and protein sidechains that reside there. The relatively slower engagement of t338 with TTR must in part be due to the steric interference of the linker branch. It is also conceivable that the tertiary amine of the linker of t338 interacts with Glu55 on the periphery of the binding site in the encounter complex. The further slowing of the binding of t338 to TTR between pH 8 and 6.5 may in part be due to titration of these interactions as the pH shift has no impact on the binding of mds84. The calculated pKa of the tertiary amine of the t338 linker is 8.3 and it would develop more positive charge at pH 6.5.

The ability to reach a stable intermediate reaction point in solution in defined conditions implied that this state might be imaged in the crystal by soaking in ligand and that all molecules in the crystal lattice might arrive as a cohort in the same state. The complexity of the weak difference electron density following short soaking times did not resemble a fully occupied binding site and most likely represents a superposition of intermediate states along the binding path.

The crowded thread‐path through the centre of the channel of the tetramer TTR implies that some accommodation by the protein must be required in order for the bulky chlorinated aryl ring to pass. This paradox of a required conformational change in what appears to be a rigid target might be resolved if the incoming ligand is able to induce a reversible relaxation of the structure at the core of the tetramer. Some evidence for this is provided by the main chain and sidechain displacements close to the binding channel observed with a fully bound molecule of t338 that have no wider impact on the protein structure. Furthermore, NMR investigations of binding of mds84 and tafamidis indicate chemical shift perturbations remote from the binding site [15]. The ability of CD to distinguish the two states of binding and in particular acceleration of State 2 from a preformed encounter complex by raising the temperature lends further credence to a role for plasticity within the binding channel. The extent of these changes and lack of reversibility, however, are likely to be very pronounced due to suboptimal features in the architecture of t338.

In summary we envisage that one bivalent ligand head group binds to the hormone binding pocket with modest affinity in the reverse mode, displacing water molecules and enabling interaction of the ligand carboxylate with mainchain and sidechain groups in the vicinity of Ser117 that projects into the binding channel and location of a chlorine atom in the outer halogen binding pocket. It is notable that in all of the soaking experiments that we have attempted with either ligand and irrespective of the interpretability of the electron density in the binding channel, the best fit of Tyr116 to the electron density by real space refinement in COOT is only achieved with strained geometric parameters. When the TTR is substantially occupied with t338, it is clear that two side‐chain conformations for Tyr116 are present with good geometry. This leads us to suspect that perturbation of Tyr116 is the likely contributor to early fluorescence and CD spectral changes as a function of time. Rapid change in protein fluorescence following excitation at 280 nm reported previously during the binding of mds84 to TTR is indicative of conformational adjustments of aromatic sidechains [12] as the ligand advances through the tetramer TTR channel. The fittings of the plots of the CD at fixed wavelength as a function of time using the exponential decay 2 (ExpDec2) function of OriginPro software revealed that the CD changes at 340 nm (*k*_1_ = 0.02 and *k*_2_ = 0.004) were faster than those at 295 nm (*k*_1_ = 0.01 and *k*_2_ = 0.001). This is associated with the ligand, first in State 1 (Figure 6A,B) entering the tunnel of the TTR tetramer till crossing to State 2. This is consistent with the positive ICD band at about 340 nm that is only due to the t388 bis‐aryl chromophore whereas the negative ICD at 295 nm also includes the CD contributions of the aromatic side‐chain residues that can sense the crossing of half of the mds38 ligand molecule through the TTR tetramer tunnel to reach State 2 (Figure 6A,B).

The results favour a remarkable threading mechanism with sequential binding and release steps and progressive inversion of the orientation of the first binding head‐group of the bivalent ligands with respect to the chiral environment of the TTR channel that is revealed by CD. Ligand‐induced relaxation of the core of the tetramer likely permits access to progressively more favourable binding interactions. The results highlight the effective complementarity of CD and x‐ray investigations and provide a prominent example of induced CD of achiral drug upon binding to a target protein.

## Author Contributions

All authors contributed to data collection, processing, analysis and manuscript preparation. S.W. led the project design.

## Funding

The authors have nothing to report.

## Ethics Statement

The authors have nothing to report.

## Consent

The authors have nothing to report.

## Conflicts of Interest

The authors declare no conflicts of interest.

## Supporting information


**File S1:** Electron density map (2mFo‐DFc blue contours, difference map in green) showing ligand t338 soaked into preformed crystals of transthyretin at pH 6.5 for 2 weeks, at 20°C.

## Data Availability

The data that support the findings of this study are available from the corresponding author upon reasonable request.
