# Supplementary material for: Induced Circular Dichroism From the Binding of Achiral Bivalent Ligands to Transthyretin
Source: J Mol Recognit. 2026 Apr 24;39:e70037. doi: 10.1002/jmr.70037 (PMC13109678; doi:10.1002/jmr.70037)
Supplement: Supplementary file 1 — File S1: Electron density map (2mFo‐DFc blue contours, difference map in green) showing ligand t338 soaked into preformed crystals of transthyretin at pH 6.5 for 2 weeks, at 20°C. [file JMR-39-e70037-s001.docx]

Supplementary S1


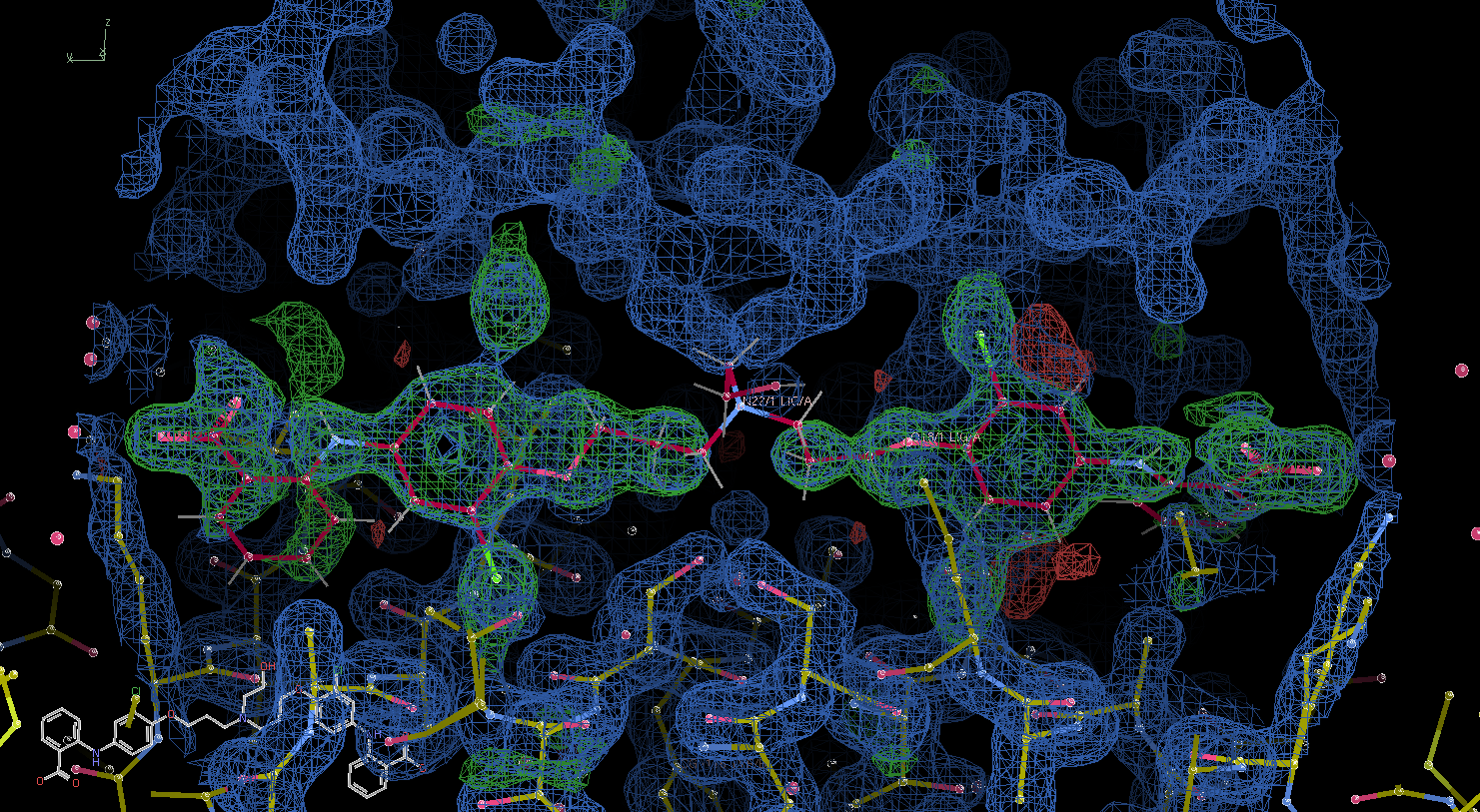


Electron density map (2mFo-DFc blue contours, difference map in green) showing ligand t338 soaked into preformed crystals of transthyretin at pH6.5 for 2 weeks, at 20^o^C. The data was collected to 1.48Å resolution at beamline i04 Diamond Light Source. Structure solution followed the same path as described for co-crystallisation in the main text but the model was subject to only one round of restrained refinement (10 cycles of refmac5) with water addition (Rw/Rfree = 19.2/23.8). The ligand was not included in the refinement and was fitted to the difference density by real space refinement in coot. Only one symmetry related copy of the ligand (red carbons) is displayed for clarity. This provides clear evidence that the ligand can gain access to the TTR tetramer in preformed crystals with sustained order.

Data

Space Group P2_1_2_1_2 a=85.93, b=44.17, c=66.02

42960 unique reflections

Rmeas 0.06(1.41), CC1/2 1(0.5), multiplicity 12, mean I/sigI 19 (1.5), completeness 99.8%. Values in brackets are for the highest resolution shell
